# Sequencing and Characterization of Pseudomonas aeruginosa phage JG004

**DOI:** 10.1186/1471-2180-11-102

**Published:** 2011-05-14

**Authors:** Julia Garbe, Boyke Bunk, Manfred Rohde, Max Schobert

**Affiliations:** 1 Institute of Microbiology, Technische Universität Braunschweig, Spielmannstr. 7, 38106 Braunschweig, Germany; 2 Current Address: Department of Clinical Sciences, Division of Infection Medicine, Biomedical Centre B14, Sölvegatan 19, 22362 Lund, Sweden; 3 HZI. Helmholtz Centre for Infection Research, Inhoffenstr. 7, 38124 Braunschweig, Germany

## Abstract

**Background:**

Phages could be an important alternative to antibiotics, especially for treatment of multiresistant bacteria as e.g. *Pseudomonas aeruginosa*. For an effective use of bacteriophages as antimicrobial agents, it is important to understand phage biology but also genes of the bacterial host essential for phage infection.

**Results:**

We isolated and characterized a lytic *Pseudomonas aeruginosa *phage, named JG004, and sequenced its genome. Phage JG004 is a lipopolysaccharide specific broad-host-range phage of the *Myoviridae *phage family. The genome of phage JG004 encodes twelve tRNAs and is highly related to the PAK-P1 phage genome. To investigate phage biology and phage-host interactions, we used transposon mutagenesis of the *P. aeruginosa *host and identified *P. aeruginosa *genes, which are essential for phage infection. Analysis of the respective *P. aeruginosa *mutants revealed several characteristics, such as host receptor and possible spermidine-dependance of phage JG004.

**Conclusions:**

Whole genome sequencing of phage JG004 in combination with identification of *P. aeruginosa *host genes essential for infection, allowed insights into JG004 biology, revealed possible resistance mechanisms of the host bacterium such as mutations in LPS and spermidine biosynthesis and can also be used to characterize unknown gene products in *P. aeruginosa*.

## Background

Opportunistic pathogens such as *Pseudomonas aeruginosa *are a major health concern due to increased antibiotic resistance [1,2]. Phages could be an alternative to antibiotics, therefore, it is important to investigate phage biology and phage-host interactions [3,4].

Phages are ubiquitous and up to 2.5*10^8 ^virus particles have been enumerated per ml in natural water [5] with about 100 million estimated phage species [6]. In August 2010, 586 complete genome sequences of phages were available and among these sequences were 46 sequences of *Pseudomonas *specific phages (National Center for Biotechnology Information; http://www.ncbi.nlm.nih.gov/; Bethesda, USA). It was stated that about 75% of all sequenced viral genes share no identity to any gene in databases, therefore, most of the viral diversity is uncharacterized [7]. The amount of sequence information of tailed phages increased dramatically in the last years [8]. Characterization of phages is based on morphology as well as on combined genomic and proteomic approaches [9-12]. Other publications describe the host range of phages, which is important with regard to phage therapy [13-15].

In this work, we characterized a newly isolated *P. aeruginosa *broad-host-range phage named JG004 on genome level and applied a transposon mutagenesis approach of the respective host bacterium to identify genes in *P. aeruginosa*, which are essential during phage infection. This approach is fast, provides new insights into phage biology and can be easily adapted for the characterization of other phages.

## Results and discussion

### Family affiliation

**Figure 1:**
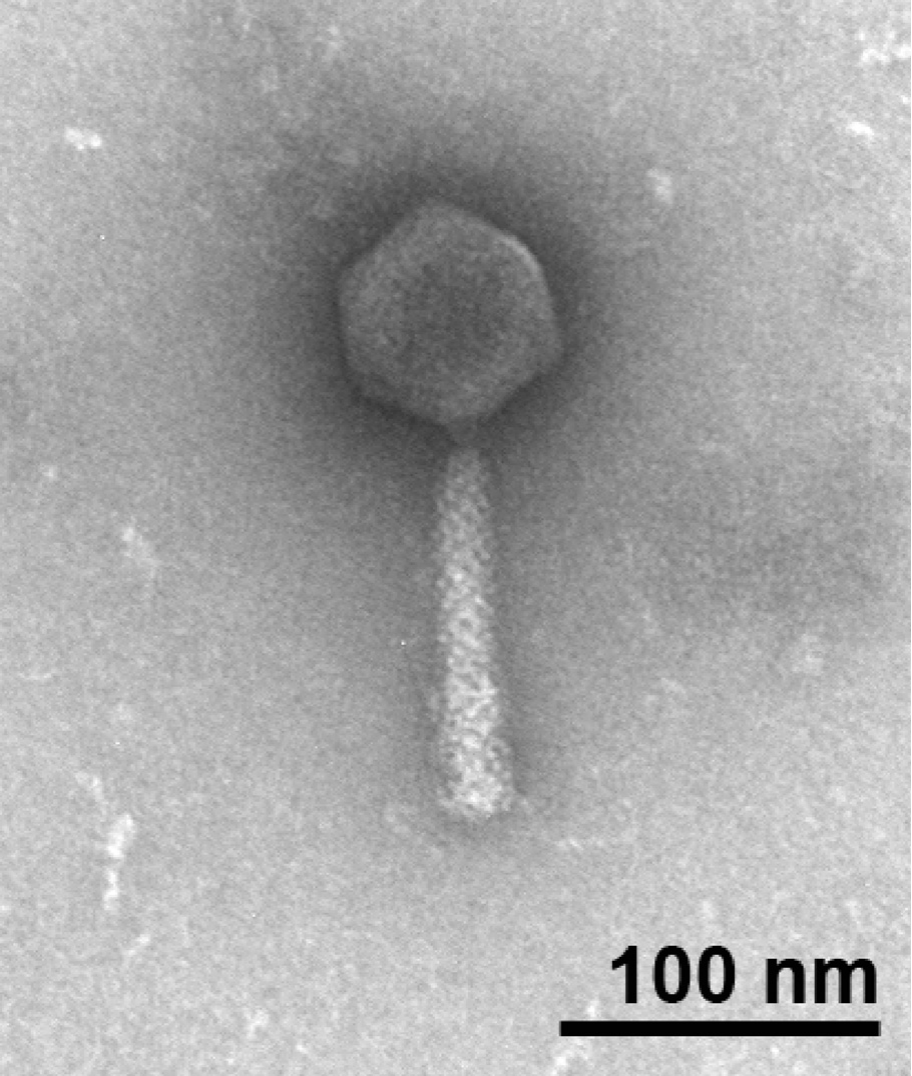
**Morphology of phage JG004**. Electron microscopic image of negatively stained phage JG004, which exhibits a contractile sheath and a central tube with a length of 115 nm and a hexagonal head structure with a diameter of 67 nm.

The morphology and size of JG004 phage particles were assessed by transmission electron microscopy (Figure 1), see Methods. In Figure 1, a isometric head structure is visible with a diameter of 67 nm. The contractile tail, which consists of a neck, a contractile sheath and a central tube, has a length of 115 nm. Due to the morphology and the identification of dsDNA by the sensitivity of restriction endonucleases like *Hind*III (data not shown), JG004 belongs to the familiy *Myoviridae*. The tailed phages comprise three families: *Myoviridae*, *Siphoviridase *as well as *Podoviridae*. It was stated that 96% of the investigated phages belong to the tailed phages. In particular, there are approximately 499 tailed *Pseudomonas *phages known, among them 139 from the family *Myoviridae *[9]. We describe the morphology of phage JG004 together with the comparison of its genome sequence below.

### Phage receptor and host range

Determination of the phage receptor was performed as described in the Methods section. Different *P. aeruginosa *mutant strains which lack flagella (Δ*fliM*), pili (Δ*pilM*) or a complete LPS (Δ*algC *) were used to investigate the ability of JG004 to infect these mutant strains. The gene *algC *encodes an enzyme with phosphoglucomutase and phosphomannomutase activity and is required for the biosynthesis of the complete *P. aeruginosa *LPS core [16]. The phage JG004 is able to lyse flagella and pili mutants but not the *algC *mutant defect in LPS biosynthesis, which indicates that LPS is the receptor of JG004.

In order to determine the host range of JG004, we used a set of 19 clinical isolates to investigate the ability of JG004 to infect these strains (Table 1). JG004 is able to infect around 50% of the tested clinical isolates (Table 1), suggesting that JG004 belongs to the broad-host-range phages. Additionally, JG004 is even capable of infecting a *P. aeruginosa mucA *mutant, which produces large amounts of exopolysaccharides and displayes a mucoid phenotype [17]. Mucoid *P. aeruginosa *strains are frequently isolated from patients suffering from cystic fibrosis and are correlated with a poor prognosis [18].

**Table 1:** Strains and phages used in this study.

Bacterial strain or phage	Phenotype or genotype	Reference
PAO1*	Wild type	[54]
PA14	Wild type	[55]
PAO1 ΔmucA*	PAO1 *mucA::aacC1-gfp *Gm^R^	Sabrina Thoma, this laboratory, unpublished
PAO1 ΔpilA*	*pilA *inactivated by allelic displacement; tagged with eGFP, Tc^R^, Gm^R^	[56]
PAO1 ΔfliM *	*fliM *inactivated by allelic displacement; tagged with eGFP, Tc^R^, Gm^R^	[56]
PAO1 Δ*algC*	*algC::aacC1-gfp *Gm^R^	Julia Garbe, this laboratory, unpublished
BT2, BT72, BT73, RN3, RN43, RN45*, NN84	Clinical CF isolates	Medical Highschool Hannover, Germany
PACF15, PACF21*, PAKL1,	Clinical CF isolates	Gerd Döring,
PAKL4*, PACF60*, PACF61*, PACF62, PACF63*		Tübingen, Germany
Nr. 18*, 19*, 26*, 29	Urinary tract infection isolate	Michael Hogardt, München, Germany
JG004	Wild type PAO1 LPS-specific lytic bacteriophage	This study

* = strains infected by phage JG004 in the host range and receptor studies.

Abbreviations: Gm^R^, resistant to gentamicin; Tc^R^, resistant to tetracyclin; eGFP, enhanced green fluorescent protein; LPS, lipopolysaccharide.

### Growth characteristics

**Figure 2:**
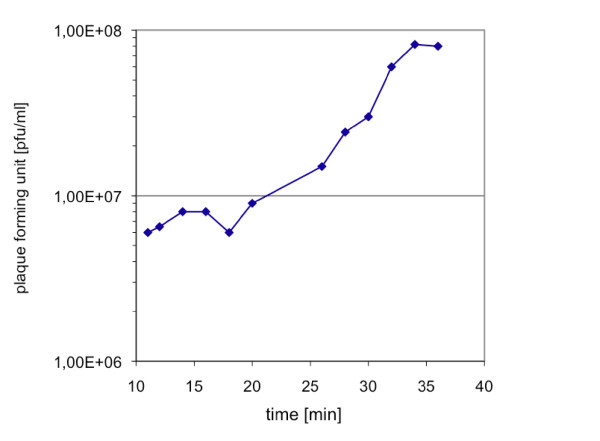
**Growth of JG004**. One step growth curve of phage JG004. A representative growth experiment of three independent experiments is shown. Within 34 min, the phage is able to produce about 13 phage progeny per infected cell.

Figure 2 shows the one step growth curve of phage JG004. The burst size, which describes the average number of phages liberated per bacterial cell as well as the latent phase were calculated as described in Methods. JG004 is able to produce approximately 13 progeny phages per cell and has a latent phase of 31 min.

### Genome properties and organization

The entire genome sequence of phage JG004 was determined as described in Methods and revealed a genome with a size of 93,017 bp. Detailed inspection of the 454 sequence reads as well as two possible genome assemblies, revealed some interesting features of the JG004 genome. A small part (bases from position 1 to 1238) of the JG004 genome has a twice to three times higher coverage by sequence reads compared to the rest of the genome (Additional file 2, Figure S1). This high coverage could be either an artifact of 454 sequencing or it indicates that this region might be present in multiple copies in the genome as a repetitive sequence. One possible arrangement could be a linear genome, which is flanked with the genome region (bases from 1 to 1238) at both ends. This is supported by the identification of 116 reads, which start exactly at the same position (position 1 in our submitted sequence; Additional file 2, Figure S2). Also, at the end of this part (position 1238), we identified 55 sequence reads which all stop at the same position indicating the endpoint of a linear genome (Additional file 2, Figure S3). This data suggests that the 1238 bp fragment is present at the beginning and the end of the genome.

To verify whether this part of the genome is present in one or multiple copies and to assess the chromosomal structure, we amplified this part of the genome by PCR using primers which bind outside of the putative repetitive sequence at the respective 5' and 3'-flanking regions. Assuming a circular genome we amplified the region using a primer which binds at position 1279 (primer 2; Additional file 2, Figure S4) and one primer which binds at position 92971 (primer 5; Additional file 2, Figure S4). Both primers generated a PCR product of 1300 bp, which corresponds to only one copy of the genome region 1 to 1238, confirming the 454 sequence data (Additional file 2, Figure S4). Moreover, we sequenced the PCR product and again confirmed the 454 sequence data. This result only indicates that the JG004 genome does not contain two consecutive copies of the putative repetitive sequence. The investigation of the linearity of the JG004 genome following treatment with exonuclease *Bal31 *[19], which degrades only double-stranded linear DNA, gave inconsistent results for the genome of JG004. We decided to integrate only one copy of the region from position 1 to 1238.

Annotation of the JG004 sequence identified 161 putative coding sequences and a GC content of 49.26% (Table 2; Additional file 1, Table S1). The general characteristics of the phage genome are summarized in Table 2.

**Table 2:** General features of the JG004 genome

Feature	Genome JG004
Genome size	93,017 bp
G+C content (G+C content host)	49,26% (68%)
No. of predicted CDSs	161
Predicted tRNAs	tRNA^Glu^; tRNA^Phe^; tRNA^Gly^; tRNA^Pro^; tRNA^Asn^; tRNA^Cys^; tRNA^Asp^; tRNA^Ile^; tRNA^Leu^; tRNA^Lys^; tRNA^Arg^; tRNA^Gln^
% of genome with non-coding regions	11.3%

The presence of genes coding for tRNAs was investigated using the tool tRNAscan-SE 1.21 [20]. With this software, we were able to identify twelve tRNAs in the genome of JG004, which are summarized in Table 2 and Additional file 1, Table S1. The presence of tRNA genes is common in members of the *Myoviridae *phages with large genomes. It was pointed out earlier that tRNA genes in phages are almost always clustered and that they may facilitate a more rapid overall translation rate, especially the translation rate of rare codons [21].

We also searched the JG004 genome for the presence of promoters, terminators and regulatory elements as described in the Methods section. No convincing sigma 70-dependent promoter region was identified in a suitable location using the web service SAK [22]. However, we identified 16 putative rho-independent terminator regions using the TransTermHP software tool [23] (Table 3). All terminators are at the right location downstream of an annotated gene. We also scanned 100 bp of the 5' region of all JG004 ORFs for the presence of conserved motifs using the program MEME [24]. We identified a conserved putative Shine Dalgarno sequence with the consensus AAGGAG (G/A)(A/T) 3-10 nt in front of the predicted ATG start codon of 108 ORFs. This sequence is more closely positioned to the ATG start codon than the Shine Dalgarno sequence in Gram-negative bacteria as e.g. *E. coli*, which is positioned 7-14 nt to the ATG start. Moreover, we detected two AT rich motifs in front of 6 and 4 CDS, respectively, which may indicate putative phage promoters (Additional file 1, Table S2).

**Table 3:** Predicted Terminator sequences.

Position	Gene	Sequence	Strand	Score
1682 - 1711	gene 3	GCGTGGTAAAGAGAA GCCCCGGG-CAGC GAAA GCTGATCCCGGGGC TTTTTTATTGCCTTG	plus	100

1711 - 1682	gene 4	CAAGGCAATAAAAAA GCCCCGGGATCAGC TTTC GCTG-CCCGGGGC TTCTCTTTACCACGC	minus	93

5477 - 5462	gene 12	GCGTTGAAAAAGAAA GAGGGC TTTC GCCCTC TGCTGGTATCTAGAG	plus	100

14969 - 14951	gene 30	ACCAAGTGATATAAA GCCCGCC CACAA GGCGGGC TTCTTTGTCTAAGGA	minus	95

31234 - 31251	gene 64	TGCGTAAAGACTTCA GGGAGGC TTCG GCCTCCC TTTCGTCGTAGGAGG	plus	93

35839 - 35864	gene 71	TATGCCACATCGACG GGGAGCTGCCT TAAC GGGTGGCTCCC TTTGTTGTTTCTGGA	plus	95

51300 - 51330	gene 91	AAAACAAGAATAATT AAGCCCCGG-AAGC GAAA GCTTGCCGGGGCTC TTTGTTATGGGTTTT	plus	100

51328 - 51302	gene 92	AACCCATAACAAAGA GCCCCGGCAAGC TTTC GCTT-CCGGGGC TTAATTATTCTTGTT	minus	95

51302 - 51328	gene 91	AACAAGAATAATTAA GCCCCGG-AAGC GAAA GCTTGCCGGGGC TCTTTGTTATGGGTT	plus	100

66578 - 66593	gene 116	CAGTTCTAACCCAAG GGGAGC TTCG GCTCCC TTTTTCATTGGAGAT	plus	100

72492 - 72507	gene 129	GCTTCAATAAGATAA GGGAGC TTCG GCTCCC TTTATTGTATCAAAG	plus	93

76657 - 76683	gene 133	GCATGTAAAATCATT GGCCCGG-GGCT TGAC AGCTTCCGGGCC TTTGTGTATTCTGAG	plus	95

79632 - 79650	gene 142	GACGCCACACTTTCA GCCCGCC CACAA GGCGGGC TTCTTTTTGCCTGAA	plus	100

80739 - 80756	gene 143	CATTATTTTAGAATT GCCCGGC GAGA GCCGGGC TTTTTCGTGGCAGGG	plus	100

87753 - 87785	gene 162	AATGCTGTAAAATAA TGCCCGTTAGGC TGAAATAAT GCTTGACGGGCA TTTTTGTATCTGTAG	plus	100

92215 - 92198	gene 173	TCTTTCCTATGAGAG GCCCCGG TCAC CCGGGGC TTGTTACGGATTGAT	minus	93

Terminator sequences are shown as displayed by TransTermHP. Each terminator sequence starts with 15 nt of the 5' tail (underlined) followed by the 5' stem, loop, 3' stem and 15 nt of the 3' tail (underlined). Score as provided by TransTermHP, only terminators with a score above 90 are shown.

### Features of the JG004 genome

**Figure 3:**
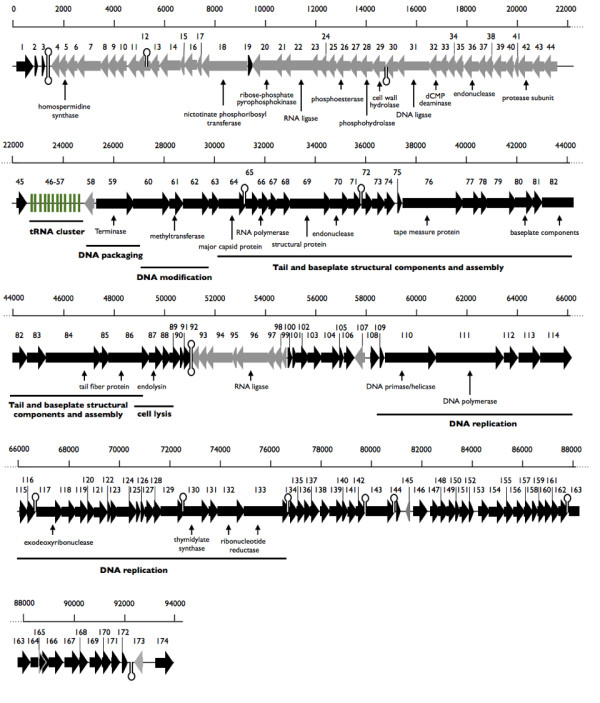
**Genome of JG004**. Schematic representation of the JG004 genome with its assumed tRNAs, genes and some functional assignments. The arrowheads point in the direction of transcription. Gene 46-57 represent the tRNAs of phage JG004. Predicted terminator structures are indicated as hairloop structures.

A schematic representation of the genome, with its predicted CDSs, the tRNA locations, some functional assignments and overall genetic organization is shown in Figure 3 and Additional file 1, Table S1. The genome of phage JG004 shows 11.3% intergenic space. This is comparable with the genome of the host *P. aeruginosa *PAO1 which has 10.6% non-coding regions [25]. Putative functions could be assigned to only 30 (18.5%) genes based on sequence similarities (Figure 3). Although phage JG004 and PAK-P1 share strong similarities, we found 19 genes with no similarities to PAK-P1 including 13 genes with no significant similarities to any protein in the database. The proteins with no similarity to other proteins are small proteins with a size between 47 aa and 112 aa. It is still difficult to accurately predict short genes with computational methods [26], therefore, these predictions are uncertain.

No significant match to proteins annotated as integrase, repressor or transposase was found, suggesting that this phage is a virulent phage which is in concordance with the results of the highly related phage PAK-P1 [27].

Gene 66 has similarities to RNA polymerases (e-value: 6e^-41^) suggesting that the phage JG004 is probably not dependent on the host transcriptional machinery. Moreover, genes encoding for enzymes of the DNA replication machinery were found, suggesting that the DNA replication is also independent from the host. We found genes with similarities to a DNA polymerase (gene 111; e-value: 0.0), a DNA helicase/primase (gene 110; e-value: 0.0), a thymidylate synthetase (gene 130; e-value: 6e^-70^), a ribonucleoside-diphosphate reductase (gene 132, 133; e-values: 0.0) and to a putative exodeoxyribunuclease (gene 117; e-value: 1e^-28^). A terminase like gene (gene 59; e-value: 0.0) could also be detected. Phage terminases are DNA packaging enzymes and are among the most conserved proteins found in phages. Some terminases also contain endonuclease activity to cut DNA into the genome length of the respective phage [28]. Two putative endonucleases were also detected (gene 36, 70; e-values: 2e^-8^, 3e^-14^). Endonucleases could be involved in the DNA packaging process or in host nucleic acid damaging. Interestingly, the putative endonuclease gene 70 has no homologue in phage PAK-P1.

Moreover, one putative methyltransferase was found (gene 61; e-value: 4e^-8^). Methyltransferases are important for the methylation of DNA to protect the DNA against it's own endonucleases or endonucleases of the host, which serve as a protection against foreign DNA and infection of phages.

Sequence based predictions identified only six genes probably involved in virion morphogenesis: gene 84 and 86 (putative tail fiber proteins; e-values: 2e^-153^; 1e^-105^), gene 80 and 82 (putative baseplate components; e-values: 2e^-63^; 2e^-95^), gene 69 (putative structural protein; e-value: 1e^-93^) as well as gene 64, which encode for the major capsid protein (e-value: 0.0). A putative tape measure protein was also detected (gene 76; e-value: 9e^-20^) close to the putative structural proteins. It was shown for phage T4 that the so called tape measure protein regulates the length of the phage tail [29].

Lysis of phages with dsDNA is accomplished by two proteins, an endolysin, which degrades the peptidoglycan and a holin, which permeabilizes the cytoplasmic membrane to release the endolysin into the periplasm [30]. We found one gene, which shares 98% identity to the endolysin of the *Pseudomonas *phage PaP1 (gene 87; 6e^-102^). However, we could not detect any similarities to a holin. This is not unexpected, since holins are very diverse and classified into twelve unrelated orthologous groups [30].

58 putative small proteins with less than 100 amino acids were found in in the genome of phage JG004. None of these small proteins has a predicted function. It was shown before that phage genomes contain small proteins with unknown function [31-33]. It is speculated that these proteins may have a role as accessory factors that bind to and subtly modify the specificity of host proteins so that they function appropriately during phage infection [34].

Interestingly, one hypothetical protein shared a low identity (32%; e-value: 0.32) with a homospermidine synthase (gene 5). We could show that phage JG004 is spermidine-dependent since it is not able to infect a *P. aeruginosa *mutant with a defect in spermidine synthesis (Table 4; see paragraph transposon mutagenesis). A homospermidine synthase produces homospermidine out of spermidine and putrescine. It is suggested that polyamines like spermidine are important for the DNA charge balance during DNA packaging [35]. The negative charge of the DNA is shielded by the positive charge of the polyamine and allows compact packaging.

**Table 4:** Transposon mutants screened with the LPS specific phage JG004.

Transposon mutant	Integration of transposon	Product name*	Pathway and Literature
TM3	*speD *(PA0654)	S-adenosylmethionine decarboxylase proenzyme	Polyamine biosynthesis (PseudoCyc)

TM4	PA0534	conserved hypothetical protein	-

TM7	PA0421	hypothetical protein	-

TM12	*wzz2 *(PA0938)	hypothetical protein	Involved in O-antigen chain length determination/LPS biosynthesis [57]

TM13	PA2555	probable AMP-binding enzyme	-

TM15	*algC *(PA5322)	phosphomannomutase	LPS biosynthesis; alginate biosynthesis [16]

TM16	*migA *(PA0705)	alpha-1,6-rhamnosyl-transferase	LPS outer core biosynthesis [42]

TM17	*rmlA *(PA5163)	glucose-1-phosphate thymidylyltransferase	LPS biosynthesis [39]

TM18	*waaL *(PA4999)	O-antigen ligase	LPS biosynthesis [58]

TM19	PA5001	hypothetical protein	LPS biosynthesis [40]

TM20, TM22	*rmlB *(PA5161)	dTDP-D-glucose 4,6-dehydratase	LPS biosynthesis [40]

TM21	PA2200	conserved hypothetical protein	-

TM23	*wapR *(PA5000)	alpha-1,3-rhamnosyl-transferase WapR	LPS outer core biosynthesis [42]

Integration of the transposon was identified by arbitrary PCR.* = according to http://www.pseudomonas.com

### Similarities of JG004 to other phages

Comparison of the genome with other phage genomes present in databases revealed that phage JG004 is highly related to the recently published phage PAK-P1 [27] with 87% identity on the nucleotide level. A Mauve analysis [36] between JG004 and PAK-P1 identified only few insertions or deletions, see Additional file 2, Figure S5. This suggests that these phages could belong to the same genus within the *Myoviridae *family.

Using BlastP searches we identified predicted proteins with a sequence identity between 43 to 99% to *Pseudomonas *phage KPP10 proteins [13] (Additional file 1, Table S1). Although phage KPP10 shares a similar morphology to JG004 with a head size of 72 nm and a tail length of 116 nm, genome alignments revealed that only 8% of the KPP10 genome shares similarities between 66% and 95% to JG004. Clearly, despite some morphological similarities, the genome sequence does not indicate any close relationship. In addition to phage PAK-P1 and to a lesser extent to phage KPP10, no significant genome sequence homology to other phages has been observed. The major capsid protein of JG004 shares 100% identity to the major capsid protein of PAK-P1 and as described by Debarbieux et al. [27], 33% identity to the major capsid protein of the *Salmonella *phage FelixO1 [27]. While JG004 and FelixO1 seem related regarding the size of phage head and tail structures (FelixO1 head: 70 nm, tail 138 nm) we did not detect any significant similarity to phage FelixO1 or related phages as *Erwinia *phage phiEa21-4 or *Enterobacteria *phage WV8 on the genome level. However, we identified four proteins with an identity from 27 to 49% to another orphan phage: *Escherichia *phage rv5 with no apparent relative [37]. Again no significant similarity on the genome level was observed. The same observation was made for the widespread PB1-like phages 14-1, F8, LBL3, LMA2, PB1 and SN. Although the phages have a similar morphology (head diameter: 74 nm; tail length: 140 nm; [38]), the genomes of these phages share no significant similarity to phage JG004.

### Transposon mutagenesis

We screened a random *P. aeruginosa *transposon library to identify *P. aeruginosa *genes essential for infection by phage JG004. A mixture of random transposon *P. aeruginosa *mutants were infected by phage JG004 (see Material and Methods). Only *P. aeruginosa *mutants, which contained a mutation in a gene essential for phage infection, survived the phage treatment. These strains were isolated and the genomic position of the mariner transposon was identified by arbitrary PCR (Table 4). A total of approximately 30000 transposon mutants were screened and 14 phage resistant mutants were isolated and analyzed. Since two mutants, TM20 and TM22 are defect in the same gene, *rmlB*, a total of 13 genes was identified, which are essential for phage infection.

The transposon screen revealed genes important for LPS biosynthesis (see Table 4 for details) like the gene *algC *which is needed for a complete LPS core in *P. aeruginosa *[16]. It also revealed the genes *rmlA *and *rmlB*, which are involved in the biosynthesis of the LPS core sugars [39,40]. These findings confirm that the phage JG004 uses LPS as receptor. Other identified genes involved in LPS biosynthesis are *wzz2*, *waaL*, *migA*, PA5000 and PA5001 (Table 4) [40].

**Figure 4:**
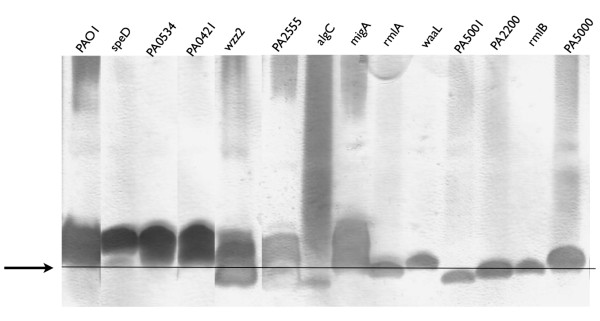
**LPS profile of transposon mutants**. Silver stained SDS-PAGE illustrating the isolated LPS of the wild type PAO1 and the transposon mutants. Only the gene, interrupted by the transposon of the respective mutant is indicated on top of the lanes, PAO1 is the *P. aeruginosa *wild type. The arrow points to the black line in the lower part of the gel. This line indicates the migration of wild type lipid A and core sugars of the LPS [42]. As indicated, the LPS of the *speD*, PA0534, PA0421, PA2555 and *migA *mutant strains appears similar to wild type LPS. The LPS profile of the remaining mutant strains is different and indicates an altered LPS structure. Interestingly, the biochemical analysis of LPS indicates that gene PA2200 might be involved in biosynthesis or modification of *P. aeruginosa *LPS due to altered migration.

Since nine out of 13 identified genes encoded proteins involved in LPS biosynthesis, we additionally isolated LPS from all mutant strains and analyzed it by electrophoresis (see Materials and Methods). Figure 4 shows the LPS profiles of the transposon mutants. The lipid A, which migrates furthest due to its size, is seen as a dark grey spot at the end of the gel. The migration depends on changes in the LPS composition, mostly in the core polysaccharide which is adjacent to the lipid A [41]. Not all LPS biosynthesis genes cause changes in the LPS which are visible by electrophorsis e.g. *migA *[42], which appears as wild type LPS. The black line in Figure 4 indicates the migration level of the wild type lipid A. Dramatic changes in the LPS profile which differs clearly from the *P. aeruginosa *wild type LPS can be seen for the *algC*, the *wzz2 *and the PA5001 mutant. Further analysis of the LPS for example Western blot analysis with antibodies specific to the different components of the LPS could provide a better understanding of the mutants, but was not involved in this phage characterization study.

We also identified genes essential for phage infection, which encode proteins of unknown function. The gene PA0421 encodes a protein with a weak similarity to an amine oxidase, the gene PA0534 encodes again a conserved hypothetical protein with an FAD binding domain, PA2555 encodes a putative acetate-CoA ligase and gene PA2200 encodes a conserved hypothetical protein with an EAL domain, which indicates that this protein could hydrolyze the signal molecule cyclic-di-GMP (http://www.pseudomonas.com). However, the role of these proteins during phage infection is unclear and is currently under investigation in our laboratory.

The gene PA0654 encodes the SpeD protein, an S-adenosylmethionine decarboxylase, which is an essential part of the spermidine biosynthesis pathway in *P. aeruginosa *[43]. These results suggest that the infection process of phage JG004 is dependent on spermidine. As pointed out earlier, JG004 also possesses a probable homospermidine synthase, which uses spermidine and purtrescine to synthesize homospermidine. Spermidine itself or homospermidine could be important substances essential for compact packaging of phage DNA by balancing the negative charge of the DNA [35].

The analysis of *P. aeruginosa *transposon mutants resistant to phage infection confirmed that phage JG004 recognizes LPS as receptor. Moreover, this approach revealed details of phage JG004 biology, e.g. its dependance on spermidine.

## Conclusions

We characterized a *P. aeruginosa *specific broad-host-range phage which is a member of the *Myoviridae *phage family. JG004 has a contractile sheath and a central tube with a length of 115 nm and an isometric head structure with a diameter of 67 nm. JG004 uses LPS as receptor and has a burst size of 13 phage particles. Genome analysis revealed that this phage shares 87% identity to phage PAK-P1. Despite its morphological similarity to other phages, no significant identity to other phage genomes was detected.

We used a transposon mutagenesis approach of the host to identify genes important for phage infection. This approach indicated a dependance of JG004 on spermidine production of the host bacterium and confirmed LPS as host receptor. In addition to the characterization of host-phage biology, this approach could be an interesting tool to perform host receptor studies or to investigate genes of unknown function such as e.g. *P. aeruginosa *genes involved in LPS biosynthesis.

## Methods

### Bacterial strains

The bacterial strains used in this study are listed in Table 1. *P. aeruginosa *strains were routinely grown in Luria Bertani (LB) broth medium aerobically at 37°C.

### Transposon mutagenesis

Transposon mutagenesis was performed with the mariner transposon as previously described [44] with the following modifications. After incubation of the mating mixture, the cells were scraped and resuspended in 1 ml LB. For selection of *P. aeruginosa *strains resistant to phage infection, the cells were incubated with a ten fold excess of the phage JG004 for 30 min at 37°C. The cells were plated on LB medium containing 200 * μ*g/ml gentamicin and 10 * μ*g/ml chloramphenicol for the inhibition of the *E. coli *S17λ*pir *strain. The insertion of the transposon was identified by arbitrary PCR and sequencing as described previously [45].

### Phage Isolation

Phages were isolated from sewage following a simple enrichment procedure. Samples from a sewage plant Steinhof in Braunschweig, Germany were centrifuged for 5 min at 4100 × g (Biofuge Fresco, Heraeus). Ten ml of the supernatant was mixed with 5 ml of a *P. aeruginosa *overnight culture and incubated in 50 ml LB broth at room temperature. After an incubation of 48 h, the cells were sedimented by centrifugation at 4100 × g (Biofuge fresco) for 10 min and the supernatant was transferred to a clean tube. To kill the remaining bacteria, several drops of chloroform were added to the supernatant and the emulsion was mixed for 30 s. To separate the phages, appropriate dilutions of the phage lysate were spotted onto bacterial lawns of top-agar plates. Top-agar plates were produced by adding approximately 5 × 10^8 ^cells/ml of *P. aeruginosa *from an overnight LB broth to 3.5 ml of LB top-agar (0.75%). The inoculated top-agar was overlaid on an LB agar plate and allowed to solidify. After incubation at 37°C for 10 to 16 h, zones of lysis were monitored. Single plaques, derived from a single phage, were separated by stinging with a pipette tip into the plaque followed by resuspending the phages in SM buffer (100 mM NaCl, 8 mM MgSO_4_, 50 mM Tris-HCl, pH 7.5). Five consecutive single plaque isolates were processed for a pure culture, which was verified by electron microscopy. The resulting phage lysate was concentrated for further analysis using polyethylenglycol and stored at 4°C.

### Electron microscopy

The morphology of the phages was determined by negative staining with 2% uranyl acetate (pH 4.8) and transmission electron microscopy. Phages were allowed to absorb onto a thin carbon film, prepared on mica, from a liquid sample for different time points, washed in TE buffer (10 mM TRIS, 2 mM EDTA, pH 6.9) and distilled water. Phages were negatively stained by floating the carbon film for appr. 15 sec on a drop of 2% aqueous uranyl acetate. Then, the carbon film was picked up with copper grids (300 mesh), blotted semi-dry with filter paper (Macherey-Nagel, MN615, 90 mm, Düren, Germany) and subsequently air dried. Samples were examined in a Zeiss EM910 transmission electron microsope at an acceleration voltage of 80 kV and calibrated using 30 nm gold particles at a magnification of 63.000. Images were recorded digitally with a Slow-Scan CCD-Camera (ProScan, 1024 × 1024, Scheuring, Germany) with ITEM-Software (Olympus Soft Imaging Solutions, Münster, Germany). Brightness and contrast were adjusted with Adobe Photoshop CS3.

### Determination of host range of phage JG004

To determine the phage host range, top-agar plates with the potential host lawn were prepared. Top-agar plates were produced by adding approximately 5 × 10^8 ^cells/ml of *P. aeruginosa *from an overnight LB broth to 3.5 ml of LB top agar (0.75%). Ten * μ*l of a phage stock solution were spotted on the top-agar plate and incubated at 37°C for 12 to 16 h. After incubation, the appearance of the lysis zones at the site where the phage suspension was added, was examined. Each phage was tested against each bacterial strain in triplicate in independent experiments. The lysis was categorized as clear (+), turbid (0) and no reaction (-) as described [14].

### Phage growth characteristics

To determine phage growth characteristics, such as burst size and duration of the infection cycle, single step growth experiments were performed as previously described for phage JG024 [46]. The burst size was determined as: (phage titer at the end of the single step growth curve at time point 34 min minus phage titer at time point 11 min) divided by phage titer at time point 11 min. The latent phase was estimated at the midpoint of the exponential phase of a one step growth experiment [47,48].

### Sequencing, analysis and annotation of phage genome

To isolate phage DNA, phages were propagated in top-agar plates as described above. After growth at 37°C the plates were overlayed with 10 ml SM buffer and incubated with shaking at 4°C for 4 h. The supernatant was filtrated (0.22 * μ*m) and stored at 4°C. Phage DNA was isolated using the Qiagen (Hilden, Germany) Lambda Kit according to manufacturer's instructions. Ten ml phage lysate with a titer of at least 10^10 ^phages/ml were used to isolate up to 1 * μ*g/* μ*l pure phage DNA. Digestion with restriction endonucleases was done following the protocols of the manufacturer.

Whole genome sequencing of the phage JG024 was done at the McGill University and Génome Québec Innovation Centre (Montréal, QC, Canada) using the Genome Sequencer FLX and 454 Technology. A total of 19294 reads with an average length of 344 bases was assembled to one single contig with a 67-fold coverage. The annotation of the unknown phage genes was done by using the software GeneMark.HMM [26]. The Heuristic approach of GeneMark was used to identify genes in small genomes under 100 kb. The identified genes were compared with the NCBI ORF Finder [49]. Nucleotide sequences were scanned for homologues using the Basic Alignment Search Tool (blastx) [50]. To search for tRNA genes in the phage genome sequence, the internet tool tRNAscan-SE 1.21 [20] was used. Results were compared with the phage PAK-P1 annotation. Sequence comparison was conducted using ClustalW2 online analysis tool [51]. Investigation of the codon usage was performed using a software tool based on JCat [52]. The genome sequence as well as the annotation is deposited at GenBank (National Center for Biotechnology Information) using the following accession number: GU988610.

### Verification of genome ends

To verify the genome ends, we amplified approximately 1300 bp of the ends of the genome by PCR and sequenced the PCR products using sequencing service of GATC Biotech (Konstanz, Germany). 30 ng genomic DNA of JG004 (see above) were used as a template in a standard PCR using TrueStart Taq polymerase (Fermentas AB, Helsingborg, Sweden) and primers described in Additional file 2, Fig. S4. The PCR products were separated on 1% TAE agarose and stained with SYBR safe (Invitrogen, Darmstadt, Germany) to verify the size. Before sequencing, the PCR products were purified using QIAquick PCR purification kit (Qiagen, Hilden, Germany) according to the manufacturer's instructions.

### Isolation and analysis of LPS

LPS was isolated and analyzed by a two-buffer tricine-based SDS-PAGE system. The isolation of the LPS was performed as described previously [16]. The SDS-PAGE consists of a 4% stacking gel and a 16.5% separating gel. Before analysis by SDS-PAGE, an aliquot of the LPS sample was combined with an equal volume of 2 × sample buffer (0.2% bromophenol blue, 10% *β*-mercaptoethanol, 40% glycerol, 3.3% SDS and 100 mM Tris HCL, pH6.8) and heated to 95°C for 5 min. Before silver staining with 0.1% silver nitrate, the gels were incubated in acetic acid for 30 min. After 5 min washing in dH2O, the gels were developed in 2.5% sodium carbonate, 0.1% formaldehyde, 0.001% sodiumthiosulfate for 2-5 min. To stop the reaction, the gels were transferred into a 2% glycine, 0.5% EDTA solution.

### Identification of promoter regions, terminator structures and other motifs

The genome of phage JG004 was scanned for the presence of putative sigma 70-dependent promoter regions using the web service SAK [22]. Putative promoter regions with a score above 1 were scanned for the presence of conserved -10 and -35 regions using the Virtual Footprint software [53] and for their genomic location, orientation and vicinity to the next gene. No promoter was identified matching these criteria. Rho-independent terminator structures were identified using the TransTermHP software tool [23]. Only rho-independent terminators at the correct genomic location with a score above 90 are displayed. Definition of the score is described in [23]. The program MEME was used for identification of conserved intergenic motifs in phage JG004 [24].

## Authors' contributions

JG designed the study and performed the experiments. BB assisted with bioinformatics knowledge and reassembled the JG004 genome sequence. Electron microscopically examinations were done by MR. MS designed the study, did bioinformatic analyses and revised the manuscript. All authors read and approved the final manuscript.

## Supplementary Material

Additional file 1 **Supplementary Table S1 and S2**. S1: Genes of phage JG004 and their predicted function. S1: Predicted position of putative phage promoter. Click here for file

Additional file 2 **Supplementary Figures**. Contains Supplementary Figures S1 to S5. Click here for file
