# Supplementary material for: Sequencing and Characterization of Pseudomonas aeruginosa phage JG004
Source: BMC Microbiol. 2011 May 14;11:102. doi: 10.1186/1471-2180-11-102 (PMC3120641; doi:10.1186/1471-2180-11-102)
Supplement: Additional file 1 — Supplementary Table S1 and S2. S1: Genes of phage JG004 and their predicted function. S1: Predicted position of putative phage promoter. [file 1471-2180-11-102-S1.PDF]

Supplementary File 1.

Supplementary Tables S1-S2

Table S1 - Genes of phage JG004 and their predicted function. Genes of phage JG004 and their predicted function. Listed are the best matches using BlastP unless they are marked with *. Since phage JG004 is highly related to the phage PAK-P1 and this phage is not completely annotated yet, we used not the best but another significant match during BlastP if it provided a prediction for the protein function.

Table S1

Gene	Begin	End	Length (bp)	Length (aa)	Predicted function; best BlastP match; accession number (GenBank); e-value	% Identity (aa residues)	Feature
1	344	997	654	217	Hypothetical protein; hypothetical protein ORF_ 0001 *Pseudomonas* phage PAK_P1; ADD64958; **$6e^{-65}$**	57 % (123/217)	CDS
2	1097	1240	144	47	Hypothetical protein		CDS
3	1517	1675	159	52	Hypothetical protein		CDS
4	1996	1730	267	88	Hypothetical protein		CDS
5*	2400	1996	405	134	Hypothetical protein; homospermidine synthase *Cupriavidus taiwanensis*; CAP62682; **0.32**	32 % (32/100)	CDS
6	2812	2390	423	140	Hypothetical protein; hypothetical protein ORF_ 0004 *Pseudomonas* phage PAK_P1; ADD64961; **$2e^{-62}$**	88 % (121/138)	CDS
7	3530	2829	702	233	Hypothetical protein; hypothetical protein ORF_ 0005 *Pseudomonas* phage PAK_P1; ADD64962; **$1e^{-105}$**	87 % (194/225)	CDS
8	3811	3533	279	92	Hypothetical protein		CDS
9	4110	3799	312	103	Hypothetical protein; hypothetical protein ORF_ 0006 *Pseudomonas* phage PAK_P1; ADD64963; **$3e^{-54}$**	100 % (103/103)	CDS
10	4496	4110	387	128	Hypothetical protein; hypothetical protein ORF_ 0007 *Pseudomonas* phage PAK_P1; ADD64964; **$1e^{-53}$**	80 % (96/120)	CDS
11	5114	4740	375	124	Hypothetical protein; hypothetical protein ORF_ 0009 *Pseudomonas* phage PAK_P1; ADD64966; $1e^{-63}$	97 % (120/124)	CDS
12	5586	5083	504	167	Hypothetical protein; hypothetical protein ORF_ 0010 *Pseudomonas* phage PAK_P1; ADD64967; **$3e^{-90}$**	98 % (162/167)	CDS
13	5884	5573	312	103	Hypothetical protein; hypothetical protein ORF_ 0011 *Pseudomonas* phage PAK_P1; ADD64968; **$3e^{-47}$**	86 % (88/103)	CDS
14	6668	5961	708	235	Hypothetical protein; hypothetical protein ORF_ 0012 *Pseudomonas* phage PAK_P1; ADD64969; **$2e^{-94}$**	92 % (176/193)	CDS
15	7001	6852	150	49	Hypothetical protein; hypothetical protein ORF_ 0014 *Pseudomonas* phage PAK_P1; ADD64971; **$3e^{-5}$**	96 % (23/24)	CDS
16	7492	7013	480	159	Hypothetical protein; hypothetical protein ORF_ 0015 *Pseudomonas* phage PAK_P1; ADD64972; **$3e^{-87}$**	99 % (158/159)	CDS
17	7686	7489	198	65	Hypothetical protein; hypothetical protein ORF_ 0016 *Pseudomonas* phage PAK_P1; ADD64973; **$2e^{-31}$**	97 % (63/65)	CDS
18	9386	7698	1689	562	putative nictotinate phosphoribosyltransferase; putative nictotinate phosphoribosyltransferase *Pseudomonas* phage PAK_P1; ADD64974; **0.0**	99 % (552/562)	CDS
19	9385	9603	219	72	Hypothetical protein; hypothetical protein ORF_ 0018 *Pseudomonas* phage PAK_P1; ADD64975; **$1e^{-19}$**	85 % (45/53)	CDS
20	10533	9658	876	291	putative ribose-phosphate pyrophosphokinase; putative ribose-phosphate pyrophosphokinase *Pseudomonas* phage PAK_P1; ADD64976; **$7e^{-152}$**	92 % (267/291)	CDS
21	10962	10543	420	139	Hypothetical protein; hypothetical protein ORF_ 0020 and ORF_ 0021 *Pseudomonas* phage PAK_P1; ADD64977 and ADD64978; **$5e^{-67}$ and $5e^{-34}$**	90 % (124/139) and 84 % (77/92)	CDS
22*	11890	10973	918	305	putative RNA ligase/tail attachment protein; RNA ligase 1 and tail attachment protein *Escherichia* phage rv5; ABI79148; **$3e^{-42}$**	40 % (120/304)	CDS
23	12309	11902	408	135	Hypothetical protein; hypothetical protein ORF_ 0023 *Pseudomonas* phage PAK_P1; ADD64980; **$1e^{-39}$**	83 % (112/135)	CDS
24	12581	12306	276	91	Hypothetical protein; hypothetical protein ORF_ 0024 *Pseudomonas* phage PAK_P1; ADD64981; **$2e^{-44}$**	98 % (89/91)	CDS
25	12819	12583	237	78	Hypothetical protein; hypothetical protein ORF_ 0025 *Pseudomonas* phage PAK_P1; ADD64982; **$1e^{-33}$**	92 % (71/78)	CDS
26*	13386	12832	555	184	putative phosphoesterase; phosphoesterase *Chryseobacterium gleum*; EFK36996; **$2e^{-22}$**	41 % (76/188)	CDS
27	13817	13386	432	143	Hypothetical protein; hypothetical protein ORF_ 0027 *Pseudomonas* phage PAK_P1; ADD64984; **$7e^{-70}$**	89 % (127/143)	CDS
28*	14367	13807	561	186	putative phosphohydrolase; Metal dependent phosphohydrolase *Lentisphaera araneosa*; EDM25460; $2e^{-05}$	34 % (51/151)	CDS
29*	14929	14369	561	186	putative cell wall hydrolase; cell wall hydrolase *Pseudomonas* phage KPP10; BAJ09106; **$7e^{-41}$**	47 % (92/196)	CDS
30	15451	14987	465	154	Hypothetical protein; hypothetical protein ORF_ 0031 and ORF_ 0032 *Pseudomonas* phage PAK_P1; ADD64989 and ADD64988; **$6e^{-80}$ and $7e^{-56}$**	97 % (148/153) and 74 % (111/152)	CDS
31	16672	15464	1209	402	DNA ligase; DNA ligase *Pseudomonas* phage PAK_P1; ADD64990; **0.0**	96 % (385/402)	CDS
32*	17085	16669	417	138	putative dCMP deaminase; putative dCMP deaminase *Pseudomonas* phage KPP10; BAJ09110; **$6e^{-32}$**	60 % (73/123)	CDS
33	17324	17088	237	78	Hypothetical protein; hypothetical protein ORF_ 0035 *Pseudomonas* phage PAK_P1; ADD64992; **$5e^{-28}$**	86 % (63/74)	CDS
34	17546	17334	213	70	Hypothetical protein; hypothetical protein ORF_ 0036 *Pseudomonas* phage PAK_P1; ADD64993; $4e^{-33}$	100 % (70/70)	CDS
35	17812	17543	270	89	Hypothetical protein; hypothetical protein *Pseudomonas* phage KPP10; BAJ09109; **$1e^{-9}$**	44 % (37/85)	CDS
36*	18480	17821	660	219	putative HNH endonuclease; HNH endonuclease Bacteriophage T5; AAU05246; **$2e^{-8}$**	43 % (39/92)	CDS
37	18796	18482	315	104	Hypothetical protein; hypothetical protein ORF_ 0037 *Pseudomonas* phage PAK_P1; ADD64994; **$8e^{-52}$**	99 % (100/101)	CDS

Table S1

Gene	Begin	End	Length (bp)	Length (aa)	Predicted function; best BlastP match; accession number (GenBank); e-value	% Identity (aa residues)	Feature
38	19088	18810	279	92	Hypothetical protein; hypothetical protein ORF_ 0038 *Pseudomonas* phage PAK_P1; ADD64995; **$2e^{-45}$**	100 % (92/92)	CDS
39	19516	19085	432	143	Hypothetical protein; hypothetical protein ORF_ 0039 and ORF_0040 *Pseudomonas* phage PAK_P1; ADD64996 and ADD64997; **$5e^{-48}$ and $2e^{-9}$**	99 % (91/92) and 100 % (27/27)	CDS
40	19877	19584	294	97	Hypothetical protein; Orf41 *Pseudomonas* phage D3; AAF80800; **$2e^{-12}$**	67 % (35/53)	CDS
41	20059	19874	186	61	Hypothetical protein; hypothetical protein ORF_ 0042 *Pseudomonas* phage PAK_P1; ADD64999; **$9e^{-27}$**	99 % (60/61)	CDS
42*	20668	20120	549	182	putative protease subunit; ClpP ATP-dependent protease subunit *Escherichia* phage rv5; ABI79187; **$6e^{-5}$**	31 % (36/118)	CDS
43	21072	20716	357	118	Hypothetical protein; hypothetical protein ORF_ 0044 *Pseudomonas* phage PAK_P1; ADD65001; **$2e^{-46}$**	99 % (117/118)	CDS
44	21536	21069	468	155	Hypothetical protein; hypothetical protein ORF_0045 and ORF_ 0046 *Pseudomonas* phage PAK_P1; ADD65002 and ADD65003; **$9e^{-51}$ and $1e^{-13}$**	97 % (100/104) and 82 % (35/43)	CDS
45	22328	22651	324	107	Hypothetical protein; hypothetical protein ORF_ 0047 *Pseudomonas* phage PAK_P1; ADD65004; **$3e^{-52}$**	99 % (106/107)	CDS
46	22657	22727	71		tRNA-Gln; genome *Pseudomonas* phage PAK_P1; GQ422154.1; $8e^{-28}$	100 % (70/70)	tRNA
47	23036	23107	72		tRNA-Arg; genome *Pseudomonas* phage PAK_P1; GQ422154.1; $5e^{-25}$	98 % (69/71)	tRNA
48	23118	23191	74		tRNA-Lys		tRNA
49	23469	23550	82		tRNA-Leu; genome *Pseudomonas* phage PAK_P1; GQ422154.1; $9e^{-33}$	100 % (81/81)	tRNA
50	23764	23836	73		tRNA-Ile; genome *Pseudomonas* phage PAK_P1; GQ422154.1; $8e^{-23}$	99 % (71/72)	tRNA
51	23849	23924	76		tRNA-Asp; genome *Pseudomonas* phage PAK_P1; GQ422154.1; $3e^{-37}$	98 % (73/75)	tRNA
52	24337	24409	73		tRNA-Cys; genome *Pseudomonas* phage PAK_P1; GQ422154.1; $6e^{-29}$	100% (72/72)	tRNA
53	24423	24495	73		tRNA-Asn; genome *Pseudomonas* phage LUZ24; AM910650.1; $2e^{-29}$	100% (72/72)	tRNA
54	24562	24636	75		tRNA-Pro; genome *Pseudomonas* phage PAK_P1; GQ422154.1; $3e^{-27}$	99 % (73/74)	tRNA
55	24646	24718	73		tRNA-Gly; genome *Pseudomonas* phage PAK_P1; GQ422154.1; $8e^{-28}$	99 % (71/72)	tRNA
56	24728	24801	74		tRNA-Phe		tRNA
57	24811	24883	73		tRNA-Glu; genome *Pseudomonas* phage PAK_P1; GQ422154.1; $6e^{-29}$	100 % (72/72)	tRNA
58	25220	24939	282	93	Hypothetical protein		CDS
59	25342	26862	1521	506	terminase large subunit; terminase large subunit *Pseudomonas* phage PAK_P1; ADD65006; **0.0**	99 % (505/506)	CDS
60	26875	28314	1440	479	Hypothetical protein; hypothetical protein ORF_ 0050 *Pseudomonas* phage PAK_P1; ADD65007; **0.0**	99 % (478/479)	CDS
61*	28324	28794	471	156	putative methyltransferase; N6 adenine-specific DNA methyltransferase *Haliangium ochraceum*; ACY13452; **$4e^{-8}$**	34 % (39/117)	CDS
62	28791	29708	918	305	Hypothetical protein; hypothetical protein ORF_ 0052 *Pseudomonas* phage PAK_P1; ADD65009; **$1e^{-139}$**	99 % (301/305)	CDS
63	29736	30146	411	136	Hypothetical protein; hypothetical protein ORF_ 0053 *Pseudomonas* phage PAK_P1; ADD65010; **$5e^{-70}$**	99 % (135/136)	CDS
64	30191	31225	1035	344	Major capsid protein; major capsid protein *Pseudomonas* phage PAK_P1; ADD65011; **0.0**	100 % (344/344)	CDS
65	31275	31751	477	158	Hypothetical protein; hypothetical protein ORF_ 0055 *Pseudomonas* phage PAK_P1; ADD65012; **$6e^{-87}$**	99 % (157/158)	CDS
66*	31789	32202	414	137	putative RNA polymerase; putative RNA polymerase *Pseudomonas* phage KPP10; BAJ09127; **$6e^{-41}$**	60% (81/137)	CDS
67	32202	32582	381	126	Hypothetical protein; hypothetical protein ORF_ 0057 *Pseudomonas* phage PAK_P1; ADD65014; **$2e^{-61}$**	99 % (125/126)	CDS
68	32579	33142	564	187	Hypothetical protein; hypothetical protein ORF_ 0058 *Pseudomonas* phage PAK_P1; ADD65015; **$1e^{-103}$**	99 % (186/187)	CDS
69	33155	34264	1110	369	putative structural protein; putative structural protein *Pseudomonas* phage KPP10; BAJ09130; $1e^{-93}$	49 % (182/379)	CDS
70	34501	35277	777	258	putative endonuclease; endonuclease SegD *Vibrio* phage KVP40; AAQ64216; **$3e^{-14}$**	34 % (72/207)	CDS
71	35290	35814	525	174	Hypothetical protein; hypothetical protein ORF_ 0060 *Pseudomonas* phage PAK_P1; ADD65017; **$9e^{-95}$**	100 % (174/174)	CDS
72	35916	36389	474	157	Hypothetical protein; hypothetical protein ORF_ 0061 *Pseudomonas* phage PAK_P1; ADD65018; **$1e^{-83}$**	99 % (156/157)	CDS
73	36389	36868	480	159	Hypothetical protein; hypothetical protein ORF_ 0062 *Pseudomonas* phage PAK_P1; ADD65019; **$3e^{-81}$**	92 % (145/159)	CDS
74	36882	37253	372	123	Hypothetical protein; hypothetical protein ORF_ 0063 *Pseudomonas* phage PAK_P1; ADD65020; **$2e^{-61}$**	98 % (119/122)	CDS
75	37361	37513	153	50	Hypothetical protein; hypothetical protein ORF_ 0064 *Pseudomonas* phage PAK_P1; ADD65021; **$3e^{-17}$**	94 % (47/50)	CDS

Table S1

Gene	Begin	End	Length (bp)	Length (aa)	Predicted function; best BlastP match; accession number (GenBank); e-value	% Identity (aa residues)	Feature
76*	37510	39876	2367	788	putative tape measure protein; tape measure protein *Burkholderia* phage BcepNazgul; AAQ63350; 9**e**$^{-20}$	33 % (70/ 217)	CDS
77	39873	40634	762	253	Hypothetical protein; hypothetical protein ORF_ 0066 and ORF_0067 *Pseudomonas* phage PAK_P1; ADD65024 and ADD65023; **1e**$^{-135}$ **and 3e**$^{-71}$	95 % (240/253) and 67 % (167/250)	CDS
78	40640	40996	357	118	Hypothetical protein; hypothetical protein ORF_ 0068 *Pseudomonas* phage PAK_P1; ADD65025; **1e**$^{-62}$	98 % (115/118)	CDS
79	40993	41910	918	305	Hypothetical protein; hypothetical protein ORF_ 0069 *Pseudomonas* phage PAK_P1; ADD65026; **1e**$^{-163}$	98% (297/305)	CDS
80*	41907	42647	741	246	Putative baseplate protein; putative baseplate protein *Pseudomonas* phage KPP10; BAJ09140; **2e**$^{-63}$	99 % (243/246)	CDS
81	42658	43029	372	123	Hypothetical protein; hypothetical protein ORF_ 0071 *Pseudomonas* phage PAK_P1; ADD65028; **5e**$^{-63}$	96 % (117/123)	CDS
82*	43031	44494	1464	487	Putative baseplate component; putative baseplate protein *Pseudomonas* phage KPP10; BAJ09142; **2e**$^{-95}$	45 % (292/488)	CDS
83	44513	45244	732	243	Hypothetical protein; hypothetical protein ORF_ 0073 *Pseudomonas* phage PAK_P1; ADD65030; **2e**$^{-126}$	97% (235/243)	CDS
84*	45255	47330	2076	691	Putative tail fiber protein; putative tail fiber protein *Pseudomonas* phage KPP10; BAJ09144; 2e^{-153}	45 % (307/686)	CDS
85	47358	47732	375	124	Hypothetical protein; hypothetical protein ORF_ 0075 *Pseudomonas* phage PAK_P1; ADD65032; **9e**$^{-43}$	69 % (87/127)	CDS
86*	48227	49246	1020	339	putative tail fiber protein; putative tail fiber protein *Pseudomonas* phage KPP10; BAJ09146; **1e**$^{-105}$	60 % (198/334)	CDS
87*	49263	49823	561	186	putative endolysin; endolysin *Pseudomonas* phage PaP1; ACZ55949; **6e**$^{-102}$	98 % (182/186)	CDS
88	49841	50080	240	79	Hypothetical protein; hypothetical protein ORF_ 0078 *Pseudomonas* phage PAK_P1; ADD65035; **4e**$^{-32}$	89 % (74/84)	CDS
89	50067	50504	438	145	Hypothetical protein; hypothetical protein ORF_ 0079 *Pseudomonas* phage PAK_P1; ADD65036; **2e**$^{-67}$	96 % (138/145)	CDS
90	50521	50652	132	43	Hypothetical protein; hypothetical protein *Pseudomonas* phage KPP10; BAJ09149; **0.1**	59 % (18/31)	CDS
91	50642	50947	306	101	Hypothetical protein; hypothetical protein ORF_ 0080 *Pseudomonas* phage PAK_P1; ADD65037; **1e**$^{-46}$	99 % (100/101)	CDS
92	51653	51345	309	102	Hypothetical protein; hypothetical protein ORF_ 0081 *Pseudomonas* phage PAK_P1; ADD65038; **4e**$^{-25}$	55 % (56/102)	CDS
93	51984	51664	321	106	Hypothetical protein		CDS
94	52795	51986	810	269	Hypothetical protein; hypothetical protein ORF_ 0082 *Pseudomonas* phage PAK_P1; ADD65039; **8e**$^{-136}$	90 % (242/270)	CDS
95	52943	52788	156	51	Hypothetical protein; hypothetical protein ORF_ 0083 *Pseudomonas* phage PAK_P1; ADD65040; **2e**$^{-20}$	83 % (43/52)	CDS
96*	54082	52946	1137	378	Putative RNA ligase; RNA ligase *Pseudomonas* phage 20phi2-1; ABY63181; **6e**$^{-44}$	33 % (151/466)	CDS
97	54346	54113	234	77	Hypothetical protein; hypothetical protein ORF_ 0085 *Pseudomonas* phage PAK_P1; ADD65042; **0.30**	43 % (32/76)	CDS
98	54617	54384	234	77	Hypothetical protein; hypothetical protein ORF_ 0085 *Pseudomonas* phage PAK_P1; ADD65042; **4e**$^{-27}$	98 % (75/77)	CDS
99	54750	54607	144	47	Hypothetical protein; hypothetical protein ORF_ 0085 *Pseudomonas* phage PAK_P1; ADD65042; **9e**$^{-06}$	71 % (26/37)	CDS
100	54863	55057	195	64	Hypothetical protein; hypothetical protein ORF_ 0086 *Pseudomonas* phage PAK_P1; ADD65043; **3e**$^{-5}$	90 % (17/19)	CDS
101	55058	55255	198	65	Hypothetical protein; hypothetical protein ORF_ 0087 *Pseudomonas* phage PAK_P1; ADD65044; **1e**$^{-26}$	87 % (56/65)	CDS
102	55267	55752	486	161	Hypothetical protein; hypothetical protein ORF_ 0088 *Pseudomonas* phage PAK_P1; ADD65045; **3e**$^{-90}$	94 % (150/161)	CDS
103	55891	56433	543	180	Hypothetical protein; hypothetical protein ORF_ 0090 *Pseudomonas* phage PAK_P1; ADD65047; **2e**$^{-68}$	96 % (123/129)	CDS
104	56430	57086	657	218	Hypothetical protein; hypothetical protein ORF_ 0091 *Pseudomonas* phage PAK_P1; ADD65048; **4e**$^{-114}$	94 % (207/221)	CDS
105	57073	57237	165	54	Hypothetical protein; hypothetical protein ORF_ 0092 *Pseudomonas* phage PAK_P1; ADD65049; **1e**$^{-22}$	97 % (52/54)	CDS
106	57240	57542	303	100	Hypothetical protein; hypothetical protein ORF_ 0093 *Pseudomonas* phage PAK_P1; ADD65050; **2e**$^{-52}$	98 % (98/100)	CDS
107	57935	57639	297	98	Hypothetical protein; hypothetical protein ORF_ 0094 *Pseudomonas* phage PAK_P1; ADD65051; **3e**$^{-54}$	100 % (99/99)	CDS
108	58411	58656	246	81	Hypothetical protein		CDS
109	58653	58838	186	61	Hypothetical protein		CDS
110	58892	60754	1854	617	DNA primase/helicase; primase/helicase *Pseudomonas* phage PAK_P1; ADD65054; **0.0**	99 % (616/617)	CDS
111	60815	63529	2715	904	DNA polymerase; polymerase *Pseudomonas* phage PAK_P1; ADD65055; **0.0**	99 % (893/904)	CDS
112	63619	64017	399	132	Hypothetical protein; hypothetical protein ORF_ 0099 *Pseudomonas* phage PAK_P1; ADD65056; **2e**$^{-66}$	99 % (131/132)	CDS
113	64215	64931	717	238	Hypothetical protein; hypothetical protein ORF_ 0101 *Pseudomonas* phage PAK_P1; ADD65058; **9e**$^{-85}$	77 % (182/238)	CDS

Table S1

Gene	Begin	End	Length (bp)	Length (aa)	Predicted function; best BlastP match; accession number (GenBank); e-value	% Identity (aa residues)	Feature
114	65033	66037	1005	334	Hypothetical protein; hypothetical protein ORF_ 0103 and ORF_ 0102 *Pseudomonas* phage PAK_P1; ADD65059 and ADD65060 3e^{-140} and 2**e^{-171}**	81 % (271/335) and 97 % (322/334)	CDS
115	66107	66340	234	77	Hypothetical protein; hypothetical protein ORF_ 0104 *Pseudomonas* phage PAK_P1; ADD65061; **8e^{-20}**	72 % (55/77)	CDS
116	66350	66571	222	73	Hypothetical protein; hypothetical protein ORF_ 0105 *Pseudomonas* phage PAK_P1; ADD65062; **5e^{-27}**	80 % (58/73)	CDS
117*	66613	67665	1053	350	Putative exodeoxyribonuclease; exodeoxyribonuclease *Escherichia* phage rv5; ABI79160; **1e^{-28}**	27 % (99/372)	CDS
118	67662	68225	564	187	Hypothetical protein; hypothetical protein ORF_ 0107 *Pseudomonas* phage PAK_P1; ADD65064; **3e^{-99}**	95 % (176/187)	CDS
119	68222	68620	399	132	Hypothetical protein; hypothetical protein ORF_ 0108 *Pseudomonas* phage PAK_P1; ADD65065; **8e-^{71}**	98 % (129/132)	CDS
120	68641	68847	207	68	Hypothetical protein; hypothetical protein ORF_ 0109 *Pseudomonas* phage PAK_P1; ADD65066; **4e^{-20}**	67 % (45/68)	CDS
121	68844	69281	438	145	Hypothetical protein; hypothetical protein ORF_ 0110 *Pseudomonas* phage PAK_P1; ADD65067; **3e^{-62}**	83 % (119/145)	CDS
122	69278	69448	171	56	Hypothetical protein; hypothetical protein ORF_ 0111 *Pseudomonas* phage PAK_P1; ADD65068; **6e^{-21}**	89 % (48/54)	CDS
123	69381	69632	252	83	Hypothetical protein		CDS
124	69629	70408	780	259	Hypothetical protein; hypothetical protein ORF_ 0112 *Pseudomonas* phage PAK_P1; ADD65069; **2e^{-151}**	99 % (257/259)	CDS
125	70405	70587	183	60	Hypothetical protein; hypothetical protein BoklE_20735 *Burkholderia oklahomensis* EO147; ZP_02357905; **2e^{-5}**	43 % (24/56)	CDS
126	70615	70821	207	68	Hypothetical protein; hypothetical protein ORF_ 0113 *Pseudomonas* phage PAK_P1; ADD65070; **9e^{-17}**	63 % (43/69)	CDS
127	70840	71175	336	111	Hypothetical protein; hypothetical protein ORF_ 0114 *Pseudomonas* phage PAK_P1; ADD65071; **5e^{-56}**	100 % (111/111)	CDS
128	71179	71391	213	70	Hypothetical protein; hypothetical protein ORF_ 0115 *Pseudomonas* phage PAK_P1; ADD65072; **4e^{-30}**	98 % (68/70)	CDS
129	71384	72334	951	316	Hypothetical protein; hypothetical protein ORF_ 0116 *Pseudomonas* phage PAK_P1; ADD65073; **2e^{-170}**	98 % (307/316)	CDS
130*	72541	73488	948	315	Putative thymidylate synthase; thymidylate synthase *Escherichia* phage rv5; ABI79172; **6e^{-70}**	49 % (158/325)	CDS
131	73491	73835	345	114	Hypothetical protein; hypothetical protein ORF_ 0118 *Pseudomonas* phage PAK_P1; ADD65075; **3e^{-61}**	100 % (114/114)	CDS
132	73865	74911	1047	348	ribonucleoside-diphosphate reductase beta subunit; ribonucleoside-diphosphate reductase beta subunit *Pseudomonas* phage PAK_P1; ADD65076; **0.0**	99 % (344/348)	CDS
133	74904	76649	1746	581	ribonucleoside-diphosphate reductase alpha chain; ribonucleoside-diphosphate reductase alpha chain *Pseudomonas* phage PAK_P1**;** ADD65077; **0.0**	98 % (567/581)	CDS
134	76846	77094	249	82	Hypothetical protein; hypothetical protein ORF_ 0121 *Pseudomonas* phage PAK_P1; ADD65078; **9e^{-19}**	53 % (44/84)	CDS
135	77094	77327	234	77	Hypothetical protein		CDS
136	77327	77563	237	78	Hypothetical protein; hypothetical protein ORF_ 0122 *Pseudomonas* phage PAK_P1; ADD65079; **1e^{-30}**	81 % (63/78)	CDS
137	77563	77877	315	104	Hypothetical protein; hypothetical protein ORF_ 0124 *Pseudomonas* phage PAK_P1; ADD65081; **0.30**	77 % (16/21)	CDS
138	78066	78332	267	88	Hypothetical protein; hypothetical protein ORF_ 0126 *Pseudomonas* phage PAK_P1; ADD65083; **1e^{-32}**	90 % (71/79)	CDS
139	78352	78855	504	167	Hypothetical protein; hypothetical protein ORF_ 0127 *Pseudomonas* phage PAK_P1; ADD65084; **3e^{-58}**	73 % (119/164)	CDS
140	78865	79059	195	64	Hypothetical protein; hypothetical protein ORF_ 0128 *Pseudomonas* phage PAK_P1; ADD65085; **2e^{-29}**	100 % (64/64)	CDS
141	79061	79300	240	79	Hypothetical protein; hypothetical protein ORF_ 0129 *Pseudomonas* phage PAK_P1; ADD65086; **8e^{-34}**	84 % (62/74)	CDS
142	79312	79563	252	83	Hypothetical protein; hypothetical protein ORF_0130 *Pseudomonas* phage PAK_P1; ADD65087; **4e^{-39}**	98 % (80/92)	CDS
143	79732	80718	987	328	Hypothetical protein; hypothetical protein ORF_0131 *Pseudomonas* phage PAK_P1; ADD65088; **0.0**	99 % (323/328)	CDS
144	80941	81114	174	57	Hypothetical protein; hypothetical protein ORF_0132 *Pseudomonas* phage PAK_P1; ADD65089; **3e^{-24}**	95 % (54/57)	CDS
145	81717	81553	165	54	Hypothetical protein		CDS
146	81743	82216	474	157	Hypothetical protein; hypothetical protein ORF_0133 *Pseudomonas* phage PAK_P1; ADD65090; **5e^{-70}**	80 % (125/157)	CDS
147	82416	82727	312	103	Hypothetical protein; hypothetical protein ORF_0134 and ORF_135 *Pseudomonas* phage PAK_P1; ADD65091 and ADD65092; **6e^{-20}** and **3e^{-18}**	91 % (47/52) and 94 % (44/47)	CDS
148	82727	83014	288	95	Hypothetical protein; hypothetical protein ORF_0136 *Pseudomonas* phage PAK_P1; ADD65093; **1e^{-31}**	93 % (88/95)	CDS
149	83011	83259	249	82	Hypothetical protein; hypothetical protein *Pseudomonas* phage KPP10; BAJ09199; 9e^{-35}	91 % (70/77)	CDS
150	83330	83458	129	42	Hypothetical protein; hypothetical protein ORF_0137 *Pseudomonas* phage PAK_P1; ADD65094; **9e^{-16}**	100 % (42/42)	CDS

Table S1

Gene	Begin	End	Length (bp)	Length (aa)	Predicted function; best BlastP match; accession number (GenBank); e-value	% Identity (aa residues)	Feature
151	83458	83766	309	102	Hypothetical protein; hypothetical protein ORF_ 0138 *Pseudomonas* phage PAK_P1; ADD65095; $1e^{-48}$	100 % (102/102)	CDS
152	83840	83986	147	48	Hypothetical protein; hypothetical protein ORF_ 0139 *Pseudomonas* phage PAK_P1; ADD65096; $1e^{-15}$	96 % (46/48)	CDS
153	84223	84606	384	127	Hypothetical protein; hypothetical protein ORF_ 0140 *Pseudomonas* phage PAK_P1; ADD65097; $2e^{-56}$	83 % (105/127)	CDS
154	84682	85353	672	223	Hypothetical protein; hypothetical protein ORF_ 0141 *Pseudomonas* phage PAK_P1; ADD65098; $6e^{-122}$	94 % (208/223)	CDS
155	85358	85696	339	112	Hypothetical protein		CDS
156	85624	86064	441	146	Hypothetical protein; hypothetical protein ORF_ 0142 *Pseudomonas* phage PAK_P1; ADD65099; $4e^{-61}$	81 % (114/141)	CDS
157	86061	86333	273	90	Hypothetical protein; hypothetical protein ORF_ 0143 *Pseudomonas* phage PAK_P1; ADD65100; $6e^{-25}$	83 % (53/64)	CDS
158	86330	86524	195	64	Hypothetical protein; hypothetical protein ORF_ 0144 *Pseudomonas* phage PAK_P1; ADD65101; $4e^{-27}$	93 % (59/64)	CDS
159	86542	86838	297	98	Hypothetical protein; hypothetical protein ORF_ 0145 *Pseudomonas* phage PAK_P1; ADD65102; $2e^{-16}$	49 % (50/104)	CDS
160	86835	87059	225	74	Hypothetical protein; hypothetical protein ORF_ 0146 *Pseudomonas* phage PAK_P1; ADD65103; $9e^{-32}$	86 % (63/74)	CDS
161	87092	87358	267	88	Hypothetical protein; hypothetical protein ORF_ 0147 *Pseudomonas* phage PAK_P1; ADD65104; $1e^{-42}$	96 % (84/88)	CDS
162	87355	87747	393	130	Hypothetical protein; hypothetical protein ORF_ 0148 *Pseudomonas* phage PAK_P1; ADD65105; $1e^{-53}$	77 % (99/130)	CDS
163	87862	88443	582	193	Hypothetical protein; hypothetical protein ORF_ 0149 *Pseudomonas* phage PAK_P1; ADD65106; $2e^{-73}$	69 % (135/196)	CDS
164	88394	89107	714	237	Hypothetical protein; hypothetical protein ORF_ 0151 *Pseudomonas* phage PAK_P1; ADD65108; $2e^{-26}$	57 % (64/113)	CDS
165	88506	88757	252	83	Hypothetical protein; hypothetical protein ORF_ 0150 *Pseudomonas* phage PAK_P1; ADD65107; $3e^{-37}$	89% (72/81)	CDS
166	89143	89622	480	159	Hypothetical protein; hypothetical protein ORF_ 0152 *Pseudomonas* phage PAK_P1; ADD65109; $6e^{-65}$	73 % (116/160)	CDS
167	89709	90236	528	175	Hypothetical protein; hypothetical protein ORF_ 0153 *Pseudomonas* phage PAK_P1; ADD65110; $1e^{-96}$	99 % (172/175)	CDS
168	90236	90517	282	93	Hypothetical protein; hypothetical protein ORF_ 0154 *Pseudomonas* phage PAK_P1; ADD65111; $2e^{-45}$	94 % (87/93)	CDS
169	90770	91222	453	172	Hypothetical protein; hypothetical protein ORF_ 0155 *Pseudomonas* phage PAK_P1; ADD65112; $5e^{-28}$	49 % (74/152)	CDS
170	91240	91530	291	95	Hypothetical protein; hypothetical protein ORF_ 0156 *Pseudomonas* phage PAK_P1; ADD65113; $2e^{-28}$	70 % (77/96)	CDS
171	91625	91891	267	88	Hypothetical protein; hypothetical protein ORF_ 0157 *Pseudomonas* phage PAK_P1; ADD65114; $8e^{-43}$	96 % (84/88)	CDS
172	91989	92183	195	64	Hypothetical protein; hypothetical protein ORF_ 0158 *Pseudomonas* phage PAK_P1; ADD65115; $2e^{-28}$	97 % (62/64)	CDS
173	92541	92311	231	76	Hypothetical protein		CDS

Table S2 - Predicted putative phage promoter elements.

Gene	P-Value	Phage promoter sequence	Position in JG004
gene 152	6.37E-029	CTTGACAGCCTAGGCCATTCCTGTAGAATGGCCCTCAAGCAAGACAAAC	83770..83818
gene 158	1.01E-027	CTTGACAGCTTAGGCCATTCCTGTATAATGGCCCTCAAGCAAGACAAAC	86259..86307
gene 167	4.69E-026	GTTGACAGCTTGGGCCATTCGCGTAGAATGGCCCTCAAGCAAGACAAAC	89624..89627
gene 172	2.85E-024	GTTGACAGCTTGGGCCATTCCTGTAGAATGGCCATCAAGCAAGGGGATG	91911..91959
gene 154	3.05E-020	GTTGACAAGGTTCCCGCCTCCTGTAGAATGGTCAGCAAGAAAGGCGGAC	84602..84650
gene 163	1.93E-019	CTTGACGGGCATTTTTGTATCTGTAGAATAGGCCTCAAGAAAGGAAAAC	87775..87823
gene 1	3.14E-019	AAAGGTGGATAGCTGCCGCTATCAATAGATCAAAATTCACA	291..331
gene 97	2.37E-017	TGACCTCGGTTGGTTTAACAAGTAATCTATCAAATCTAACA	54408..54368
gene 96	9.01E-017	TAATCTCGGTTGGTTTGACAATTAATCTAGTAAATTTAAAA	54137..54097
gene 157	3.24E-015	TAACCTGAGCATCGGTCACAATGACGCTCTTAAGACTCTCA	85986..86026
