# Supplementary material for: Sequencing and Characterization of Pseudomonas aeruginosa phage JG004
Source: BMC Microbiol. 2011 May 14;11:102. doi: 10.1186/1471-2180-11-102 (PMC3120641; doi:10.1186/1471-2180-11-102)
Supplement: Additional file 2 — Supplementary Figures. Contains Supplementary Figures S1 to S5. [file 1471-2180-11-102-S2.PDF]

Supplementary File 2.

Supplementary Figures S1-S5

Supplementary Figure S1

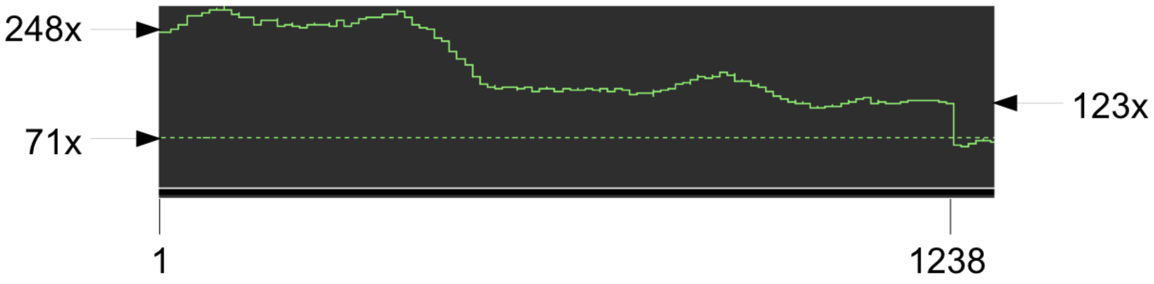

Supplementary Figure S1:

Coverage plot of JG004 genome assembly from position 1 to 1238 (Screenshot from the Interactive Visual Analytics Tool for Genome Assemblies, Amos Hawkeye, see reference below.). A circular chromosome is assumed with no repeats. The average coverage of 71 is drawn as dashed line. At putative chromosome start a coverage of more than threefold compared to the average chromosomal coverage is observed.

Schatz, M.C., Phillippy, A.M., Shneiderman, B., and Salzberg, S.L. (2007). Hawkeye: an interactive visual analytics tool for genome assemblies. Genome Biol *8*, R34.

Supplementary Figure S2

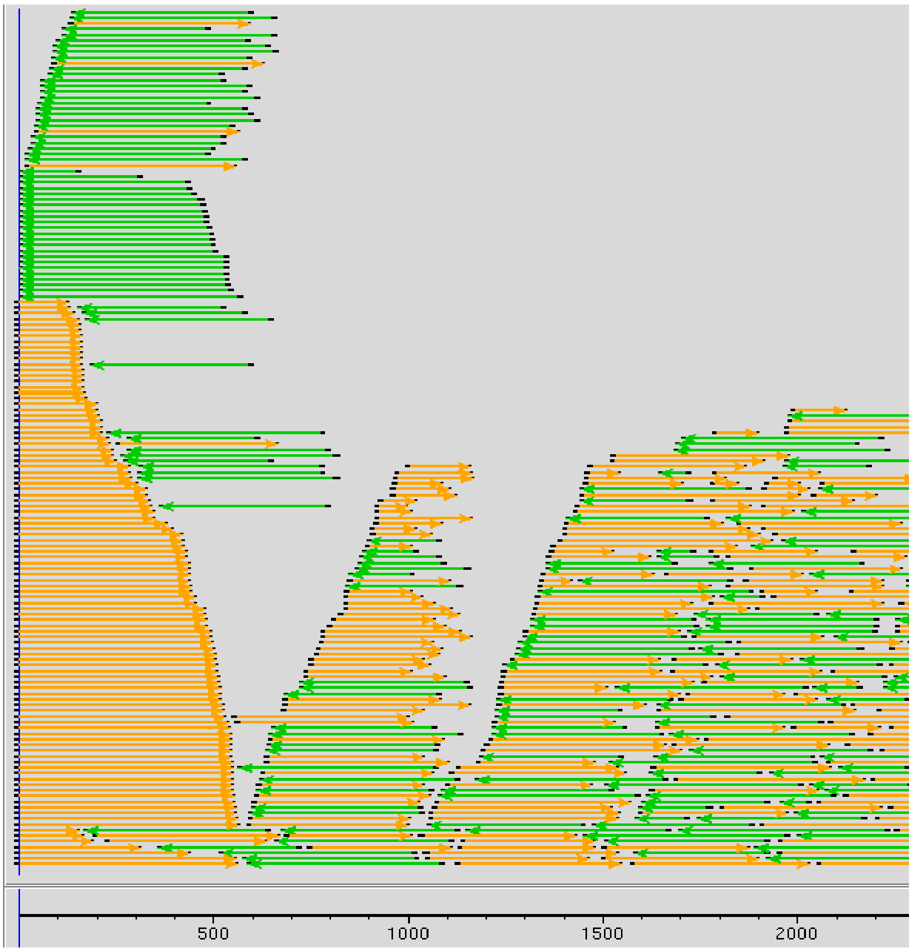

Legend Supplementary Figure S2:

Gap4 Screenshot of the Staden Package displaying assembled sequence reads of the 454 sequencing of JG004. Shown are forward (orange) and reverse (green) reads of the 5' end of JG004. Note the forward reads on the left side starting at the same position.

Supplementary Figure S3

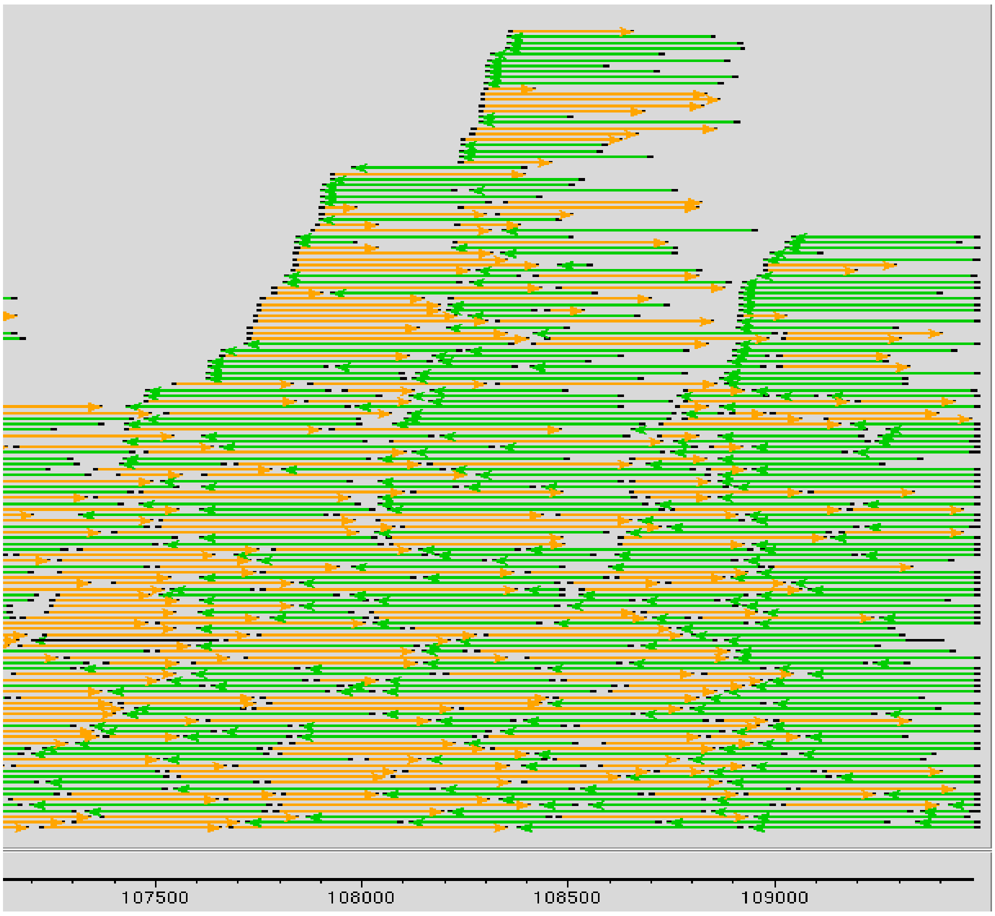

Legend Supplementary Figure S3:

Gap4 Screenshot of the Staden Package displaying assembled sequence reads of the 454 sequencing of JG004. Shown are forward (orange) and reverse (green) reads of the hypothetical 3' end of JG004. Note the reverse reads on the right side starting at the same position.

Supplementary Figure S4

A

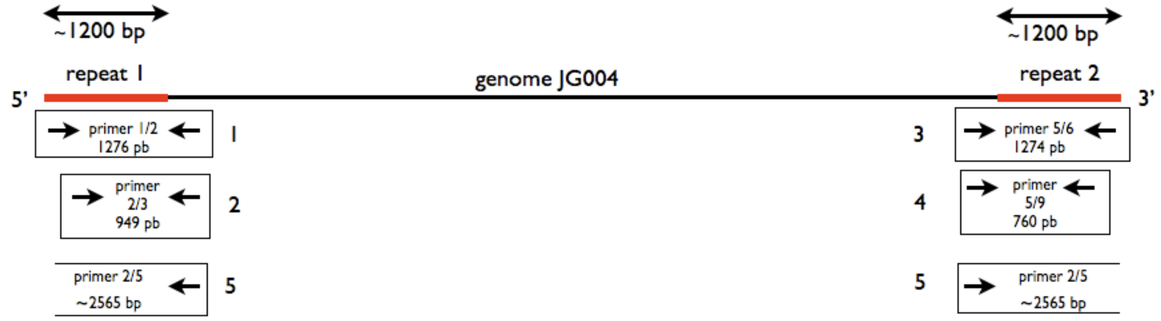

B

Primer	Sequence (5'-3')	Start position (bp)
1	GACAGCTTACACCCGCTCTC	4
2	GTTCGCCTTCTGCCAGTTAT	1279
3	ACAAGGAGACCCAATGACAA	331
5	TTGGGCCTATAACCCCTTTC	92971
6	ACCTCCGCTACCCTAACCTC	94244
9	GTGTCCTAGCTGGCTTGGG	93730

C

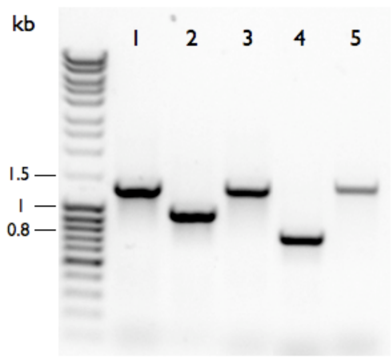

Legend Supplementary Figure S4a-c:

PCR verification of possible repeats in the JG004 genome. (A) Schematic illustration of one possible variant of the JG004 genome with the assumption that the putative repetitive region (1 to 1238) is present in two copies, labeled “repeat 1” and “repeat 2” . The parts of the genome which were amplified by PCR and sequenced are indicated as well as the used primers and the estimated size. The sequences and start positions of the primers used in this study are described in (B). (C) The sizes of the PCR products are shown on a 1 % TAE agarose gel. The numbers of the PCR reactions in (A) correspond to the numbers in (C). Note that PCR product “5” has a size of approx. 1300 bp, indicating the presence of only one copy of the repetitive region.

Supplementary Figure S5

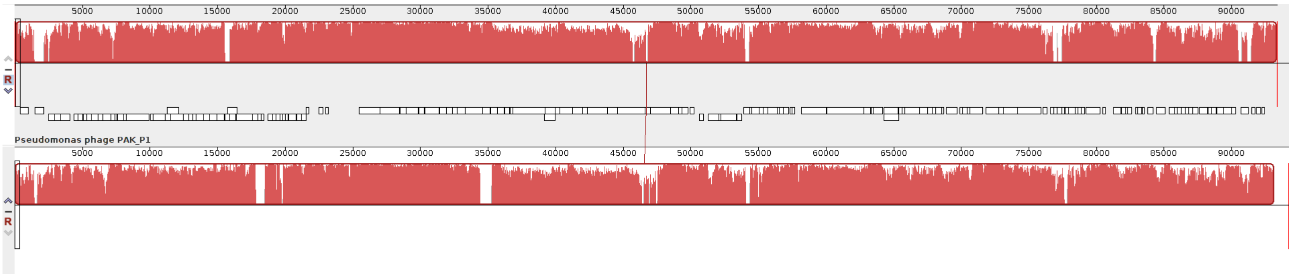

Legend Supplementary Figure S5:

Graph and schematic representation of a Mauve comparison using phage PAK-P1 and JG004 (from top to bottom). The linear genome sequences of each phage, the position of the individual Orfs and their orientation are indicated. A ruler displaying the base count in kb is on top of the respective phage genome. The red graph indicates the mutual conservation of the respective DNA sequence. A low red graph indicates low conservation. A red graph of zero indicates genome areas with insertions or deletions.
